# Quantitative evaluation of aortic valve regurgitation in 4D flow cardiac magnetic resonance: at which level should we measure?

**DOI:** 10.1186/s12880-022-00895-2

**Published:** 2022-09-27

**Authors:** Malgorzata Polacin, Julia Geiger, Barbara Burkhardt, Fraser M. Callaghan, Emanuela Valsangiacomo, Christian Kellenberger

**Affiliations:** 1 Institute of Diagnostic and Interventional Radiology, University Hospital Zurich, University of Zurich, Raemistrasse 100, 8091 Zurich, Switzerland; 2 Department of Diagnostic Imaging, University Children`s Hospital, University of Zurich, Zurich, Switzerland; 3 Division of Pediatric Cardiology, Pediatric Heart Center, University Children`s Hospital, University of Zurich, Zurich, Switzerland; 4 Center for MR Research, University Children’s Hospital, University of Zurich, Zurich, Switzerland; 5 Children’s Research Center, University Children’s Hospital, University of Zurich, Zurich, Switzerland

**Keywords:** Cardiac magnetic resonance, 4D flow, Aortic regurgitation

## Abstract

**Purpose:**

To find the best level to measure aortic flow for quantification of aortic regurgitation (AR) in 4D flow CMR.

**Methods:**

In 27 congenital heart disease patients with AR (67% male, 31 ± 16 years) two blinded observers measured antegrade, retrograde, net aortic flow volumes and regurgitant fractions at 6 levels in 4D flow: (1) below the aortic valve (AV), (2) at the AV, (3) at the aortic sinus, (4) at the sinotubular junction, (5) at the level of the pulmonary arteries (PA) and (6) below the brachiocephalic trunk. 2D phase contrast (2DPC) sequences were acquired at the level of PA. All patients received prior transthoracic echocardiography (TTE) with AR severity grading according to a recommended multiparametric approach.

**Results:**

After assigning 2DPC measurements into AR grading, agreement between TTE AR grading and 2DPC was good (*κ* = 0.88). In 4D flow, antegrade flow was similar between the six levels (*p* = 0.87). Net flow was higher at level 1–2 than at levels 3–6 (*p* < 0.05). Retrograde flow and regurgitant fraction at level 1–2 were lower compared to levels 3–6 (*p* < 0.05). Reproducibility (inter-reader agreement: ICC 0.993, 95% CI 0.986–0.99; intra-reader agreement: ICC 0.982, 95%CI 0.943–0.994) as well as measurement agreement between 4D flow and 2DPC (ICC 0.994; 95%CI 0.989 – 0.998) was best at the level of PA.

**Conclusion:**

For estimating severity of AR in 4D flow, best reproducibility along with best agreement with 2DPC measurements can be expected at the level of PA. Measurements at AV or below AV might underestimate AR.

## Background

Aortic regurgitation (AR) is a common valvular disease and can occur isolated or in the context of congenital heart disease (CHD). Although echocardiography is considered the imaging modality of choice for diagnosis and classification of AR [1, 2], flow measurements are increasingly performed as part of cardiac magnetic resonance (CMR) exams, since CMR provides highly precise information about ventricular volumes and function and is operator independent. Usually, CMR uses two-dimensional phase contrast sequences (2DPC), where regurgitant flow volume can be derived from an individually selected imaging plane. However, four-dimensional (4D) flow CMR is growingly used in the clinical setting since it enables retrospective measurements of any blood flow at any level of a particular vessel within the acquired volume [3–5]. This especially facilitates acquisition of flow measurements when anatomy is complex and placement of region of interest (ROI) planes for 2DPC flow measurements is challenging. Furthermore, 4D flow uses free-breathing technique which increases patient compliance.

There is no final recommendation regarding the optimal imaging plane for 2DPC aortic flow measurements with some authors preferring measurements near the aortic valve [6, 7] and some in the ascending aorta [8–11]. Correspondingly, the optimal level for AR measurements in 4D flow CMR still needs to be defined. The aim of the present study is to analyse aortic flow (antegrade, retrograde, net flow and regurgitant fraction) in CHD patients with AR at six different levels- from below the aortic valve to below the brachiocephalic trunk- in order to find the most accurate position for quantifying the degree of AR.

## Methods

### Patient cohort

29 patients with CHD and AR that underwent CMR between May 2018 and December 2019 in our department were retrospectively included. Prior to CMR, all patients underwent transthoracic echocardiography (TTE). The median time between TTE and CMR was 14.2 days (range 1–27 days). In 21 of the 29 patients, follow-up TTE was performed after CMR (median time 15 months 12 days). Ethical approval was obtained and all enrolled subjects (or their parent or legal guardian in the case of children under the age of 16 or illiterate participants) gave written informed consent. Demographics of the patient cohort are shown in Table 1.

**Table 1:** Patient demographics

	Patients *n* = 29
Age (years, range)	32.2 ± 16 (6–63)
Male, %	22 (76%)
BMI	24.7 ± 5
BSA	1.73 ± 0.3
LVEF (%)	53.2 ± 8
LVEDV [ml]	104.7 ± 28
RVEF (%)	50 ± 10
BAV	9
TTE AR mild	13
TTE AR moderate	13
TTE AR severe	3

*AR* Aortic regurgitation*, BAV* Bicuspid aortic valve,* BMI* Body mass index, *BSA* Body surface, *LVEF* Left ventricular ejection fraction, *LVEDV* Left ventricular end-diastolic volume, *RVEF* Right ventricular ejection fraction, *TTE* Transthoracic echocardiography

### Data acquisition

*Transthoracic echocardiography* – TTE and follow-up TTE exams were performed by a board-certified attending cardiologist from a maximum-care centre (University Children`s Hospital and University Hospital Zurich) using high-end scanners (Phillips IE 33, Philips Healthcare, Best, the Netherlands). AR severity was graded according to a recommended TTE multiparametric approach based on structural (e.g. valve morphology and LV size), qualitative (e.g. jet width, jet density and jet deceleration rate), semiquantitative (vena contracta width, end-diastolic flow velocity in the descending aorta) and quantitative (flow convergence method and quantitative pulsed Doppler) parameters [12, 13].

*CMR* – CMR exams were performed on a 1.5 T GE Discovery MR450 scanner using a dedicated 32-channel cardiac coil. As part of a standard CMR protocol, a 4-point 4D flow acquisition covering the chest from below the cardiac apex to above the anterior superior mediastinum was acquired. 4D flow was ECG gated, acquired in free breathing with axial orientation. Echo time (TE) was 2.0 ± 0.0 ms, repetition time (TR) 4.2 ± 0.1 ms, and a flip angle of 15°. Velocity encoding adjusted based upon previous TTE data: range 160–300 cms^−1^ (2.0 ± 0.5). Spatial resolution was acquired at a range of 1.8–2.8 mm in-plane resolution (median 2.3 mm), reconstructed to 1.3–1.6 mm (median 1.4) and slice thickness ranged from 1.8 to 2.6 mm (median 2.2 mm). The mean temporal resolution was 24.6 ± 6.2 ms equating to 20–25 phases per cardiac cycle. A local shim volume covering the heart and major vessels was applied and k-t acceleration parallel imaging was used (*kat-ARC* = 8). In addition to the 4D flow, 2DPC sequences were acquired in the ascending aorta using an oblique plane aligned perpendicular to the vessel axis at the level of the pulmonary bifurcation. 2DPC was acquired during breath holding with ECG gating, valve tracking and encoding velocity of 160–350 cms^−1^. Slice thickness was 4 mm, in-plane resolution of 0.9–3.3 mm (median 1.75 mm), reconstructed to 0.9–1.6 mm (median 1.3), TE: 3.6 ± 0.1 ms; TR: 6.4 ± 0.1; Flip angle: 20°. Temporal discretization was 20 intervals per cardiac cycle, giving resolution of 42.4 ± 7.7 ms.

### Data analysis

**Fig. 1:**
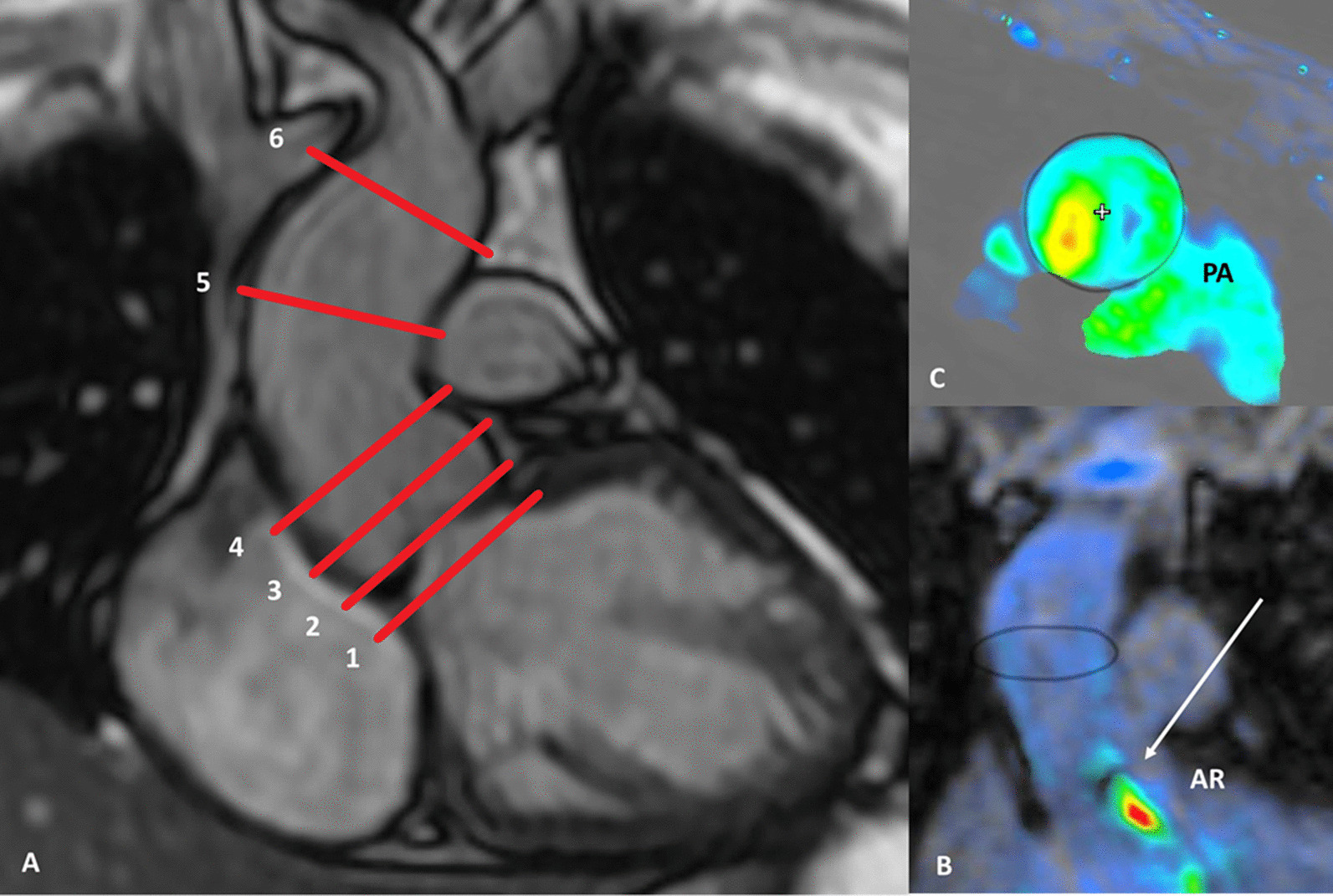
Measurement levels for aortic flow measurements. **a**: Level 1: below aortic valve, Level 2: at the aortic valve, Level 3: aortic sinus, Level 4: sinotubular junction, Level 5: ascending aorta at the level of the pulmonary arteries, Level 6: ascending aorta below the brachiocephalic trunk. **b/c:** 4D flow image at the level of the pulmonary arteries and visualization of the regurgitant aortic jet (arrow)

Two expert readers (8 years and 19 years CMR experience), blinded to each other and clinical information, used a dedicated cloud-based post-processing software (Arterys Inc., San Francisco, CA, USA) to perform measurements [5]. Aortic valve (AV) was localized in the 3D anatomical multiplanar dataset and ROIs were placed manually at six levels: (1) below the AV (2) at the AV, (3) in the aortic sinus, (4) at the sinotubular junction, (5) at the level of the pulmonary arteries (PA), and (6) below the brachiocephalic trunk (BCT) (Fig. 1). The circumferential in-plane ROIs at the aforementioned levels were tracked throughout the cardiac cycle and—if necessary- manually corrected. All six ROIs included information about antegrade flow (antegrade flow volume through the AV), retrograde flow (retrograde flow volume through the AV), net flow (antegrade plus retrograde volume) as well as the regurgitant fraction (derived from the retrograde flow and defining severity of AR). Similarly, in-plane ROIs were placed manually in 2DPC sequences (acquired at the level of the pulmonary arteries [14]), which also contained information about antegrade, net, retrograde aortic flow and regurgitant fraction. For intra-reader agreement, one reader repeated all measurements after 10 weeks. For correlation with TTE, 2DPC measurements of every patient were assigned into AR severity grades according to Spampinato et al. [9].

### Statistical analysis

Statistical analysis was performed using commercially available software (IBM SPSS Statistics, release 25.0; SPSS, Armonk, NY, USA). Statistical significance was assumed at a p-value below 0.05. Antegrade, reverse and net aortic flow volumes, and regurgitant fractions in 4D flow were compared between six levels with one-way ANOVA. Agreement between TTE AR grading and 2DPC measurements was assessed by calculating Cohen’s kappa coefficients, after transforming 2DPC measurements into AR severity grades according to Spampinato et al. [9]. Kappa coefficients were interpreted as follows: ‘poor’ < 0.50, ‘moderate’ between 0.50 and 0.74, ‘good’ between 0.75 and 0.90, and ‘excellent’ > 0.9.

Intra- and inter-reader agreement in 4D flow measurements as well as measurement agreement between CMR 2DPC and 4D flow was assessed with intraclass correlation coefficient (ICC) and Bland Altman analysis.

## Results

*Patient cohort –* From initially 29 included patients, two patients had to be excluded from further analysis due to aliasing artifacts at all levels of 4D flow measurements (both patients AR °II, bicuspid AV with combined insufficiency and stenosis). Furthermore, in four patients (two patients with AR°II and two with AR° III), aliasing occurred at the AV and was visible from level 1 to level 3; therefore, measurements from those levels were excluded. Detailed patient characteristics are listed in Table 2.

**Table 2:** Detailed patient characteristics

Congenital heart disease	*Gender*age	AR TTE (°)	BAV	Eccentric aortic jet	Aortic ectasia	CMR 2D PC (%)	CMR 4D Level 1 (%)	CMR 4D Level 2 (%)	CMR 4D Level 3 (%)	CMR 4D Level 4 (%)	CMR 4D Level 5 (%)	CMR 4D Level 6 (%)
*RV*	M, 31	1	no	no	yes	12	21	15	20	19	17	14
*SOVA*	M, 62	1	no	no	no	7	2	1	7	9	7	8
*TOF*	M, 63	1	no	yes	yes	16	14	8	9	15	24	27
*DORV*	M, 43	1	no	no	no	9	10	6	8	11	8	5
*dTGA*	M, 16	1	no	no	no	6	10	1	4	8	6	5
*TAC*	W, 46	1	no	no	no	11	9	7	9	13	11	14
*BAV*	M, 16	1	yes	no	no	16	8	2	16	17	15	9
*PAT/VSD*	W, 21	1	no	no	no	6	7	0	9	7	7	7
*TOF*	M, 39	1	no	no	yes	13	4	3	9	14	9	5
*dTGA*	M, 12	1	no	yes	no	12	16	10	13	15	15	16
*TAC*	W, 41	1	no	no	yes	15	9	4	14	20	12	20
*DCRV*	W, 28	1	no	no	no	16	1	1	10	17	17	11
*PA/VSD*	W, 51	1	no	yes	no	17	5	4	5	12	16	26
*BAV*	M, 27	2	yes	no	no	30	18	13	30	29	29	30
*CoA*	M, 39	2	yes	no	no	30	11	11	28	33	35	30
*AVSD*	M, 37	2	no	no	no	31	14	6	32	31	32	24
*dTGA*	M, 35	2	no	no	no	37	30	11	26	41	41	43
*DORV*	M, 8	2	yes	no	no	27	33	18	25	41	32	28
*TAC*	M, 6	2	no	yes	yes	23	21	8	11	32	31	33
*TOF*	M, 31	2	no	no	no	16	12	1	20	21	20	16
*Dysplastic AV*	M, 19	2	no	no	no	23	4	6	24	27	23	24
*BAV*	M, 52	2	yes	yes	no	45	10	27	36	42	44	42
*IAA*	W, 16	3	no	yes	no	48	27	12	32	46	49	52

*0* no AR due to (second) valve replacement between first TTE and follow-up TTE; *2DPC* phase contrast flow measurements*; 4D* four-dimensional flow measurement(s)*; “- “* excluded measurements due to aliasing artifacts; *AE* aortic ectasia; *AR TTE 1–3* echocardiographic grading of aortic regurgitation (1 = mild, 2 = moderate, 3 = severe)*; (B)AV (*bicuspid) aortic valve*; AVdys* dysplastic aortic valve; *AV OP* any surgery performed on aortic valve (without aortic valve replacement); *AVR* Aortic valve replacement, *CMR* Cardiac magnetic resonance*; CoA* Coarctation of the aorta*; DCRV* Double chambered right ventricle; *DORV* Double outlet right ventricle*, dTGA* Dextro-Transposition of the great arteries,* FU* follow-up,* IAA* Interrupted aortic arch, *PAT* Pulmonary atresia,* SOVA* Sinus of Valsalva aneurysm*, TAC* Truncus arteriosus communis*, TOF* Tetralogy of Fallot*, TTE* Transthoracal echocardiography*, VSD* Ventricle septal defect

**Fig. 2:**
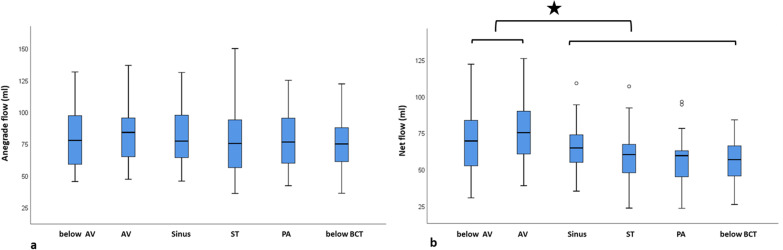
Antegrade flow and net flow in aortic regurgitation. **a**: No significant difference was measured between the six different levels regarding antegrade flow. **b**: Aortic net flow was significantly higher below or at the level of the aortic valve compared to measurements above the valve and in the ascending aorta; the highest net flow was measured at the level of the aortic valve. *AV* Aortic valve, *ST* Sinotubular junction, *PA* Pulmonary arteries, *BCT* Brachiocephalic trunk

*4D flow measurements: Antegrade flow and net flow –* No significant difference was measured between the six levels regarding antegrade flow (80.2 ml, range 36.3 – 150.2 ml; *p* = 0.866; Fig. 2a) and this was true for AR°I (*p* = 0.750) and AR°II/III (*p* = 0.918).

Net flow was significantly higher at level 1 and 2 (74.7 ml; range 30.9 – 126.1 ml) than at levels 3–6 (59.3 ml, range 23.7 – 109.2 ml; *p* = 0.001) (Fig. 2b); further analysis revealed that the difference was more pronounced in AR °II/III (*p* = 0.02) than in AR °I (*p* = 0.327).

*4D flow measurements: Retrograde flow and regurgitant fraction –* In AR°I, mean retrograde flow was significantly lower at level 1–2 (4.9 ml, range 0.3 – 15.3 ml) than at levels 3–6 (8.3 ml, range 3.2 – 17.1 ml; *p* = 0.003). Also, in AR°II/III, mean retrograde flow was lower at level 1–2 (13 ml, range 1.2 – 36.7 ml) than at levels 3–6 (30.8 ml, range 10.5 – 80.5 ml; *p* = 0.003).

**Fig. 3:**
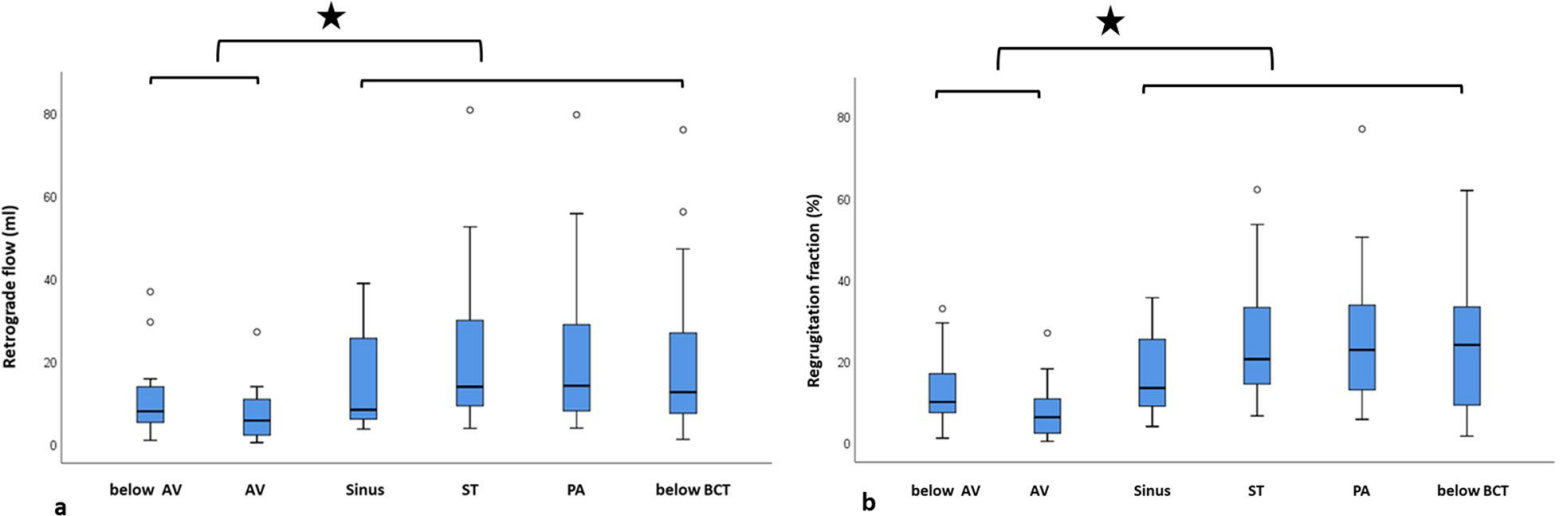
Retrograde flow and regurgitant fraction in aortic regurgitation. Retrograde flow **a** as well as regurgitant fraction **b** were significantly lower below or at the level of the aortic valve compared to measurements above the valve and in the ascending aorta (level 3–6); the lowest retrograde flow and regurgitant fraction was measured at the level of the aortic valve. *AV* Aortic valve, *ST* Sinotubular junction, *PA* Pulmonary arteries, *BCT* Brachiocephalic trunk

Similarly, in AR°I, regurgitant fraction at level 1 and 2 was 6.8% (range 0.4 – 20.5%) and at levels 3–6, 12.2% (range 4 – 26.6%; *p* = 0.001). In AR°II/III, regurgitant fraction at level 1 and 2 was 14.6% (range 1.3 – 33%) and at levels 3–6 33.9% (range 10.9 – 77.1%; *p* < 0.001). The lowest retrograde flow and regurgitant fraction was at the level of the aortic valve (Fig. 3).

*Regurgitant fraction in 2DPC and 4D flow –* Comparing 2DPC (per default acquired at the level of PA) with the six measurement levels of 4D flow, measurement agreement regarding regurgitant fraction was best at the level of the pulmonary arteries (ICC 0.994; 95%CI 0.989 – 0.998) with the narrowest limits of agreement (2.1 to − 5.2%, Table 3).

**Table 3:** Measurement of agreement between 2DPC and 4D flow level 1–6

	Measurement agreement ICC (95% CI)	Limits of agreement
Level 1 (below AV)	0.951 (0.895 – 0.977)	− 12.9 – 6.6%
Level 2 (AV)	0.957 (0.909 – 0.980)	− 14.5 – 3.8%
Level 3 (Sinus)	0.985 (0.968 – 0.993)	− 6.6 – 4.1%
Level 4 (ST)	0.952 (0.899 – 0.978)	− 12.1 – 6.7%
Level 5 (PA)	0.994 (0.989 – 0.998)	− 5.2 – 2.1%
Level 6 (below BCT)	0.907 (0.807 – 0.957)	− 23.7 – 2.7%

Measurement agreement was best at level 5, the level of the pulmonary arteries

*AV* Aortic valve, *ST* Sinotubular junction, *PA* Pulmonary arteries, *BCT* Brachiocephalic trunk

**Fig. 4:**
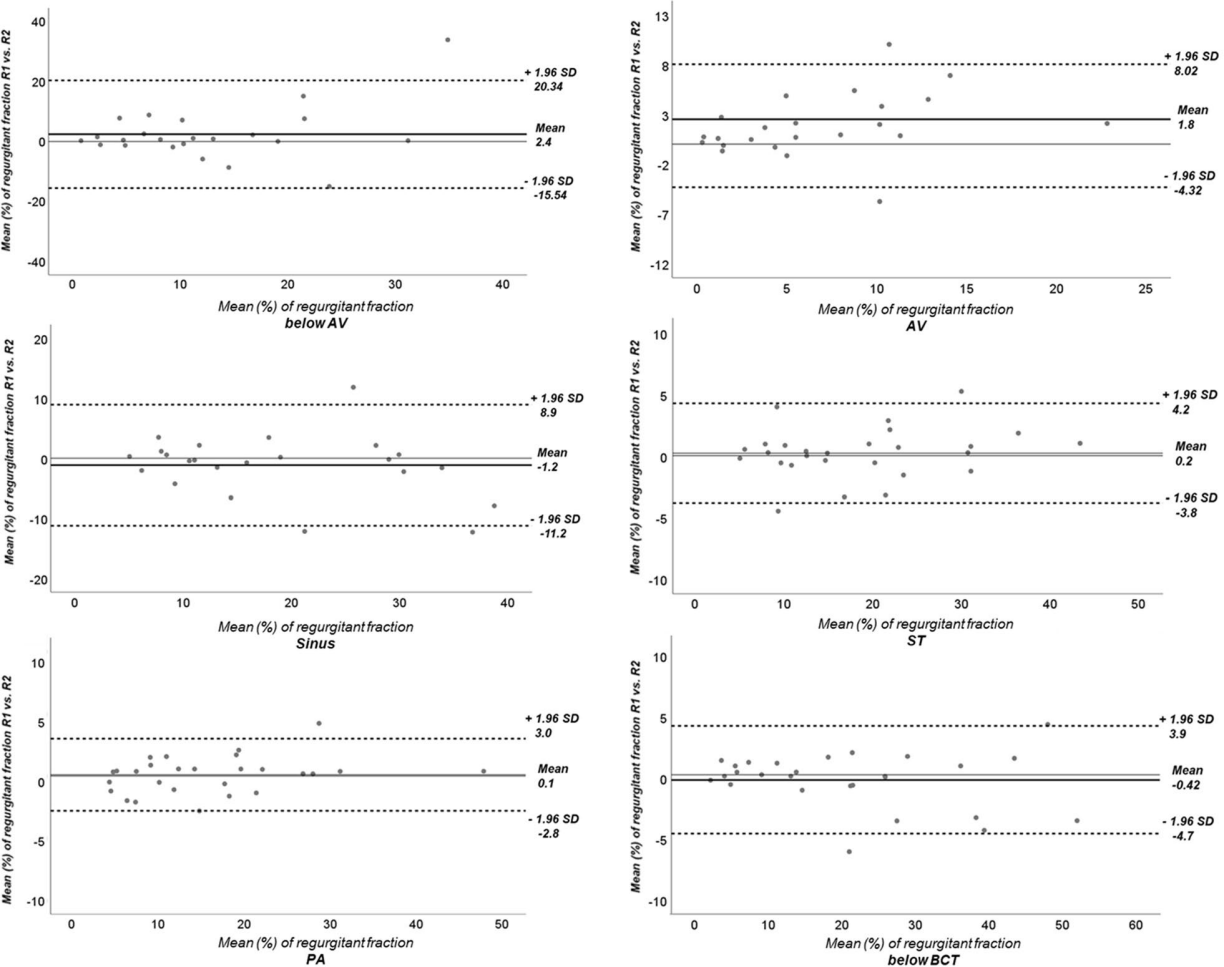
Inter-reader agreement of aortic regurgitation in level 1–6 visualized by Bland Altmann plots. Best inter-reader agreement and the narrowest limits of agreement were at the level of the pulmonary arteries. *AV* Aortic valve, *ST* Sinotubular junction, *PA* Pulmonary arteries, *BCT* Brachiocephalic trunk

*Reproducibility –* Inter-reader agreement in 4D flow measurements was best at the level of the pulmonary arteries (ICC 0.993, 95% CI 0.986 – 0.998) with the narrowest limits of agreement (− 2.8–3%) (Table 4, Fig. 4). Intra-reader agreement was also highest at the level of the pulmonary arteries (ICC 0.982; 95%CI 0.946 – 0.994) (Table 4).

**Table 4:** Inter- and intra-reader agreement of aortic regurgitation in 4D flow level 1–6

	Inter-reader agreement ICC (95%CI)	Intra-reader agreement ICC (95%CI)
Level 1 (below AV)	0.592 (0.257 – 0.802)	0.948 (0.881 – 0.977)
Level 2 (AV)	0.812 (0.533 – 0.922)	0.837 (0.644 – 0.928)
Level 3 (Sinus)	0.889 (0.759 – 0.951)	0.837 (0.643 – 0.924)
Level 4 (ST)	0.984 (0.963 – 0.989)	0.975 (0.936 – 0.991)
Level 5 (PA)	0.993 (0.986 – 0.998)	0.982 (0.946 – 0.994)
Level 6 (below BCT)	0.987 (0.971 – 0.992)	0.975 (0.923 – 0.989)

Inter-and intra-reader agreement was best at level 5, the level of the pulmonary arteries

*AV* Aortic valve, *ST* Sinotubular junction, *PA* Pulmonary arteries, *BCT* Brachiocephalic trunk

*AR grading in 2DPC and TTE –* After assignment of 2DPC measurements into AR severity according to the classification of Spampinato et al. [9], 2DPC measurements revealed the same AR grading as TTE in all patients with one exception (*κ* = 0.88). One patient with a bicuspid aortic valve and eccentric regurgitant jet was classified as moderate AR in TTE, whereas 2DPC measurements showed a regurgitant fraction of 45%. According to the aforementioned classification, a regurgitant fraction of above 42% would suggest severe AR. However, follow-up TTE revealed AR° II in this patient. No changes in TTE AR grading occurred between initial TTE and follow-up exam (available in 21 patients), except one patient that received aortic valve replacement (AVR) between both TTE exams.

## Discussion

The aim of this study was to analyse aortic flow in CHD patients with AR at six different levels—from below the aortic valve to below the brachiocephalic trunk—in 4D flow CMR with the intention to find the most accurate position for quantifying the degree of AR.

For AR quantification in 4D flow, best reproducibility along with best measurement agreement with 2DPC can be expected in the ascending aorta at the level of the pulmonary arteries.

Since CMR became gold standard for measurements of ventricular volumes und function, evaluation of valvular disease in CMR became more important. Regurgitant volume and regurgitant fraction in AR can be sufficiently measured in 2DPC [9, 15]. However, in CHD patients, choosing correct imaging planes for accurate 2DPC flow measurements within the exam can be challenging and time consuming due to complex anatomy and often multiple valvular pathologies. 4D flow CMR is incrementally used in the clinical setting since flow measurements can be retrospectively performed in any vessel within the scanned volume and complex flow patterns can be clearly visualized throughout the cardiac cycle [16]. However, there are no definite recommendations yet at which level AR grading should be performed in 4D flow.

Analyzing the antegrade volume through the aortic valve, 4D flow revealed similar volumes in all six levels. Retrograde flow and regurgitant fraction, on the other hand, were significantly lower at the level of AV or below AV (level 1 and 2) than in measurements above the valve. This observation seems to be in contradiction with some published results, which report higher regurgitant volumes when measuring at the AV and lower regurgitant volumes when measuring in the ascending aorta [8, 10]. A possible explanation is increased turbulent blood flow around the AV with consecutive signal loss, leading to underestimation of the regurgitant jet and successively AR severity [17, 18]. In fact, most patients in our CHD cohort displayed AV pathologies like fibrotic valve degeneration, postoperative changes from major heart surgeries or congenital AV diseases like a bicuspid valve, favoring complex flow patterns around the valve [14, 19, 20]. Due to lower regurgitant flow volumes in level 1 and 2, net flow was consecutively higher in level 1 and 2 compared to net flow in level 3 to level 6, especially in moderate/severe AR.

Since 2DPC flow was acquired at the level of the pulmonary arteries, it was likely that best measurement agreement between 2DPC and 4D flow regarding regurgitant fraction was at level 5, the level of the pulmonary arteries. Finally, best inter- and intra-reader agreement regarding regurgitant fraction in 4D flow was also observed at the level of the pulmonary arteries.

There is no definitive recommendation which scale should be applied for grading AR severity based on CMR 2DPC measurements. According to Spampinato et al. [9], a scale that best matches the TTE derived AR severity in 2DPC measurements would define a regurgitant fraction below 21% as AR°I, a regurgitant fraction between 22 and 41% as AR °II and a regurgitant fraction above 42% as AR° III. After transforming 2DPC measurements into the proposed AR grading scale, we observed good agreement between TTE AR grading and 2DPC. Beside one patient that received AVR between initial and follow-up TTE due to symptomatic AR °III, no differences in AR grading were detected between initial and follow-up TTE; however, follow-up TTE was only available in 21 patients.

In one out of 27 patients, AR grading differed between TTE and 2DPC: both TTE exams revealed moderate AR, while both readers measured higher regurgitant fractions in 4D flow (45%) and 2DPC (44%), rather fitting a severe AR. Especially in moderate or severe AR, defining the cut-off for correct grading between TTE and 2DPC seems challenging [15, 21, 22]. Furthermore, this patient had a bicuspid valve and an eccentric jet, both features leading to potential underdiagnosis of AR in echocardiography [19, 23, 24].

Several limitations need to be mentioned. First, this is a small, retrospective, single centre study with patients with mostly mild and moderate AR; only three patients had a severe aortic valve insufficiency. Prospective studies with more patients and an equal amount of severe AR are needed to proof the reliability of AR measurements at the level of the pulmonary arteries. Furthermore, since flow turbulences around the AV in CHD patients are suspected to cause reduced detectability of the regurgitant jet near the AV, further studies should consider comparing CHD patients with AR vs. non-CHD patients with AR only.

Although velocity encoding in 4D flow was adapted to prior TTE data, we had to exclude two patients completely due to aliasing as well as measurements at level 1–3 in four further patients—among them two patients with aortic valve replacement -, reducing an already small patient cohort. This observation shows, that predefining correct VENC in 4D flow can be challenging in clinical setting, especially when valve disease causing potential aliasing (e.g. combined insufficiency and stenosis) is not previously suspected. Finally, for true aortic valve regurgitation reverse flow to the coronaries (approx. 5% of the cardiac output) needs to be taken into consideration when measuring above the coronary sinuses [25].

## Conclusion

For estimating severity of aortic regurgitation in CMR 4D flow, best reproducibility along with best agreement with 2DPC measurements can be expected at the level of the pulmonary arteries. In patients with CHD and AR, measurements at or below the AV might underestimate AR, possibly due to complex flow patterns around the valve.

## Data Availability

The datasets generated and analysed during this study are not publicly available due to their patient referable character, thereby compromising individual privacy, but are available from the corresponding author on reasonable request.

## Abbreviations

2D: Two-dimensional
4D: Four-dimensional
BAV: Bicuspid aortic valve
AR: Aortic regurgitation
AV: Aortic valve
AVR: Aortic valve replacement
CHD: Congenital heart disease
CMR: Cardiac magnetic resonance
FU: Follow-up
LV: Left ventricle/left-ventricular
LVEDV: Left ventricular end-diastolic volume
LVEF: Left ventricular ejection fraction
min: Minutes
PC: Phase contrast
SSFP: Steady- state free precession
sec: Seconds
T: Tesla
TE: Echo time
TR: Repetition time
ROI: Region of interest
VENC: Velocity encoding
